# A Systemic embolization model in a rat with real-time micro-fluoroscopy for direct quantification of thrombolysis

**DOI:** 10.1016/j.mex.2026.104036

**Published:** 2026-07-07

**Authors:** Peter Scheer, Jana Hložková, Adam Novobilský, Jaroslav Ondruš, Eliška Brhelová, Jana Doležalová, Ahmet Davut Aksu, Radka Opatřilová, Alba Grayston, Anna Rosell, Josef Mašek, Robert Mikulík

**Affiliations:** a International Clinical Research Center, St. Anne´s University Hospital in Brno, Brno, Czech Republic; b Pharmaceutical Faculty, Masaryk University, Brno, Czech Republic; c Veterinary Research Institute, Department of Pharmacology and Toxicology, Brno, Czech Republic; d Neurovascular Research Laboratory, Institut de Recerca Hospital Vall d'Hebron, Barcelona, Spain; e Department of Neurology, Faculty of Medicine, Masaryk University, Brno, Czech Republic

**Keywords:** Thromboembolism, Barium sulfate, Micro-fluoroscopy, Ischemic stroke, Alteplase

## Abstract

Recanalization after intravenous thrombolysis remains incomplete in many stroke patients, highlighting the need for standardized preclinical methods to evaluate thrombolytic therapies. We developed a rat systemic embolization model combined with high-resolution micro-fluoroscopy enabling direct, real-time, and quantitative assessment of thrombolysis in vivo. Human fibrin-based BaSO₄ labeled clots were introduced into the abdominal aorta (three clots/animal) to assess alteplase (rtPA) efficacy at doses of 0.9 and 5 mg/kg/h. Micro-CT imaging served as a reference modality. Micro-fluoroscopy continuously visualized clot dissolution, detecting a mean of two clots per animal. Thrombolysis resulted in a dose-dependent reduction in clot area (mean ± SD; -46 ± 26% and -81 ± 22% for 0.9 and 5 mg/kg/h rtPA, respectively, versus -21 ± 14% in controls). Measurement reliability was high (ICC = 0.89), and concordance with micro-CT supported concurrent validity. The model demonstrated predictive and construct validity, and offers technically feasible, reproducible, and standardized platform for pharmacodynamic evaluation of thrombolytic agents.

• A systemic embolization model with C-arm micro-fluoroscopy enables direct, real-time visualization of thrombolysis in vivo.
• Applying multiple clots per animal reduces the total number of animals required.
• The model allows standardized, reproducible testing of thrombolytics with translational relevance.

## Graphical abstract

**Figure:**
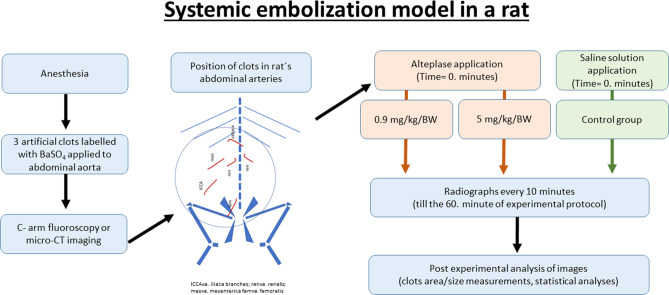

## Specifications table

**Table:** 

**Subject area**	Pharmacology, Toxicology and Pharmaceutical Science
**More specific subject area**	Thrombolysis models
**Name of your method**	Systemic embolization in vivo model with micro-fluoroscopy imaging
**Name and reference of original method**	None
**Resource availability**	*software:*https://imagej.net/ij/; https://www.graphpad.com/support/; https://www.bruker.com/en/products-and-solutions/preclinical-imaging/micro-ct/3d-suite-software.html *reagents: Alteplase (Actilyse, Boehringer-Ingelheim, Germany, batch number: 907,124), barium sulphate (Micropaque, Guerbet, Roissy CdG Cedex, France), Tisseel Kit (Baxter, USA), saline solution (Braun, Germany), ketamine (Ketamidor, ORION Pharma Animal Health, Finland), xylazine (Xylapan, Vétoquinol, France), alpha-chloralose (Sigma-Aldrich, USA), urethane (Sigma-Aldrich, USA)* *equipment: C-arm micro-fluoroscopy (Labscope, Glenbrook Technology, Inc., USA); micro-CT Skyscan 1276 (Bruker, Germany)*

## Background

For over a decade, venous and arterial thromboembolisms have been the leading causes of mortality in developed countries [1] due to myocardial infarction, cerebrovascular events, or pulmonary thromboembolism [2]. Intravenous thrombolysis is the first-choice therapy for acute ischemic stroke [3] but may also be used in acute pulmonary embolism [4] and acute myocardial infarction [5]. However, recanalization success in ischemic strokes depends on many factors (site of embolism, length of therapeutic window, used thrombolytic drug, etc.) making the overall reperfusion rate 10–47% [6], limiting the benefits of these therapies. Therefore, there is a need for better thrombolytic drugs, or combination therapies, that achieve higher recanalization rates and improve outcomes in millions of patients.

Traditionally, thromboembolic animal models were designed to mimic all aspects of human vascular occlusion stroke for understanding mechanisms of thrombus formation and pathological changes during and after the occlusion, as well as for testing and developing new thrombolytics [7]. However, translation of promising thrombolytic strategies from experimental studies to clinical practice remains rare: e.g.of >1.000 preclinical studies in acute stroke, only two have reached clinical application [8]. There are several important limitations that constrain the translational value of current experimental thromboembolic models, including middle cerebral artery occlusion (MCAO) models. First, thrombolytic efficacy is typically assessed indirectly through downstream endpoints such as infarct volume or neurological deficit [9], rather than through direct, real-time quantification of clot dissolution. This limits the ability to evaluate pharmacodynamic properties of thrombolytic agents and to distinguish between partial and complete recanalization processes [10]. Second, many models do not allow continuous in vivo visualization of thrombolysis, relying instead on endpoint or intermittent assessments, which obscures the temporal dynamics of clot lysis [9]. Finally, commonly used imaging approaches lack sufficient temporal resolution [11] or practicality for dynamic, whole-body monitoring of thrombolysis [12], limiting their utility for standardized drug testing [8], reproducibility and comparability of results [10]. A major contributing factor has been insufficient methodological quality in preclinical studies, leading to variable internal validity, inflated effect sizes, and biased conclusions, thereby undermining the justification and design of subsequent clinical trials [8].

Here, we present a novel systemic thromboembolic animal model (SE) designed to address these limitations by enabling direct, real-time, and quantitative assessment of thrombolysis *in vivo*. In this model, thrombolytic efficacy of alteplase (recombinant tissue plasminogen activator, rtPA) is measured on multiple artificial clots using high-resolution micro-fluoroscopy, allowing continuous visualization of clot dissolution and precise characterization of thrombolysis dynamics. This approach provides a direct pharmacodynamic readout of thrombolytic activity, overcoming reliance on indirect outcome measures. The use of multiple clots per animal increases experimental efficiency while enabling robust quantitative analysis. To validate imaging accuracy, measurements obtained by micro-fluoroscopy are compared with X-ray micro-computed tomography (micro-CT). In addition, to explore applicability in cerebral circulation, the Circle Of Willis Branches Occlusion (COWBOi) model with micro-CT imaging was used to evaluate rtPA efficacy on the same artificial clots at selected treatment doses (described in Supplementary Material only).

## Method details

The SE model was evaluated using a comprehensive validation framework including predictive validity, construct validity, concurrent validity, and internal validity [13].

Predictive validity was assessed by evaluating whether the thrombolytic effects of alteplase correspond to its known pharmacological profile in humans. A clinically relevant dose (0.9 mg/kg/h) [14] and a supra-therapeutic dose (5 mg/kg/h) [15] were administered, and treatment effects were quantified using relative change in clot area, lysis rate, and area under the curve (AUC) in comparison to saline controls. The model was considered to demonstrate predictive validity if alteplase induced a statistically significant, dose-dependent thrombolytic effect with temporal dynamics consistent with clinical observations [13].

Construct validity was evaluated by assessing whether the model captures the intended biological process of thrombolysis through coherent and consistent changes across multiple quantitative endpoints (clot size reduction, lysis rate, and AUC) [16].

Concurrent validity was assessed by comparing thrombolytic measurements obtained using micro-fluoroscopy with those obtained using micro-CT, a reference imaging modality [17], to determine agreement between methods.

Because the thrombolytic efficacy of alteplase was assessed using a novel imaging approach, internal validity criteria were also incorporated, including protocol simplicity, reproducibility under standardized conditions, inter-observer reliability (independent blinded analysis), randomization, controlled experimental design, and standardized data processing procedures [13].

In addition, model sensitivity (ability to detect treatment effects and dose-response relationships) and feasibility (clot detectability, procedural success rate, and safety) were evaluated to support its applicability as a pharmacodynamic screening platform.

• *Experimental design*

In SE model, 51 rats received three artificial clots (each 10 mm in size, see below) into the abdominal aorta. Animals were randomly assigned (simple randomization by drawing lots by the animal caretaker) for micro-fluoroscopy and micro-CT imaging, respectively. For both imaging methods, rats were also randomized to three groups: a) treated with intravenous thrombolysis using rtPA at a dose of 0.9 mg/kg/hour (0.9 rtPA treated; n = 10 for micro-fluoroscopy and n = 7 for micro-CT) and 5 mg/kg/hour (5 rtPA treated; n = 10 for micro-fluoroscopy and n = 7 for micro-CT) with an initial bolus of 10% volume and 90% infusion for 60 min (a flow rate 1.3–1.5 ml/hour); and b) control group (n = 10 for micro-fluoroscopy and n = 7 for micro-CT) that received an intravenous saline solution in an equivalent volume, manner, and time. The thrombolytic therapy was monitored for 60 min by a C-arm micro-fluoroscopy system (Labscope, Glenbrook Technology, Inc., USA; resolution of 20 line pairs per millimeter, a radiation dose of 0.35 mSv/hour) or the micro-CT Skyscan 1276 (Bruker microCT, Belgium; voltage 100 kV, current of 200μA, exposure time 125 ms). Projective images (radiographs) were obtained from multiple perspectives every 10 min.

To test the ability of rtPA to lyse the artificial clots in conditions of brain ischemia, the COWBOi model with micro-CT imaging was included. Description of COWBOi model with results is in the Supplementary Material.

The exclusion criteria set for the experiment were death during anesthesia, surgical procedures (performed before clot administration), and the inability to detect an artificial clot.

• *Clot preparation*

**Fig. 1:**
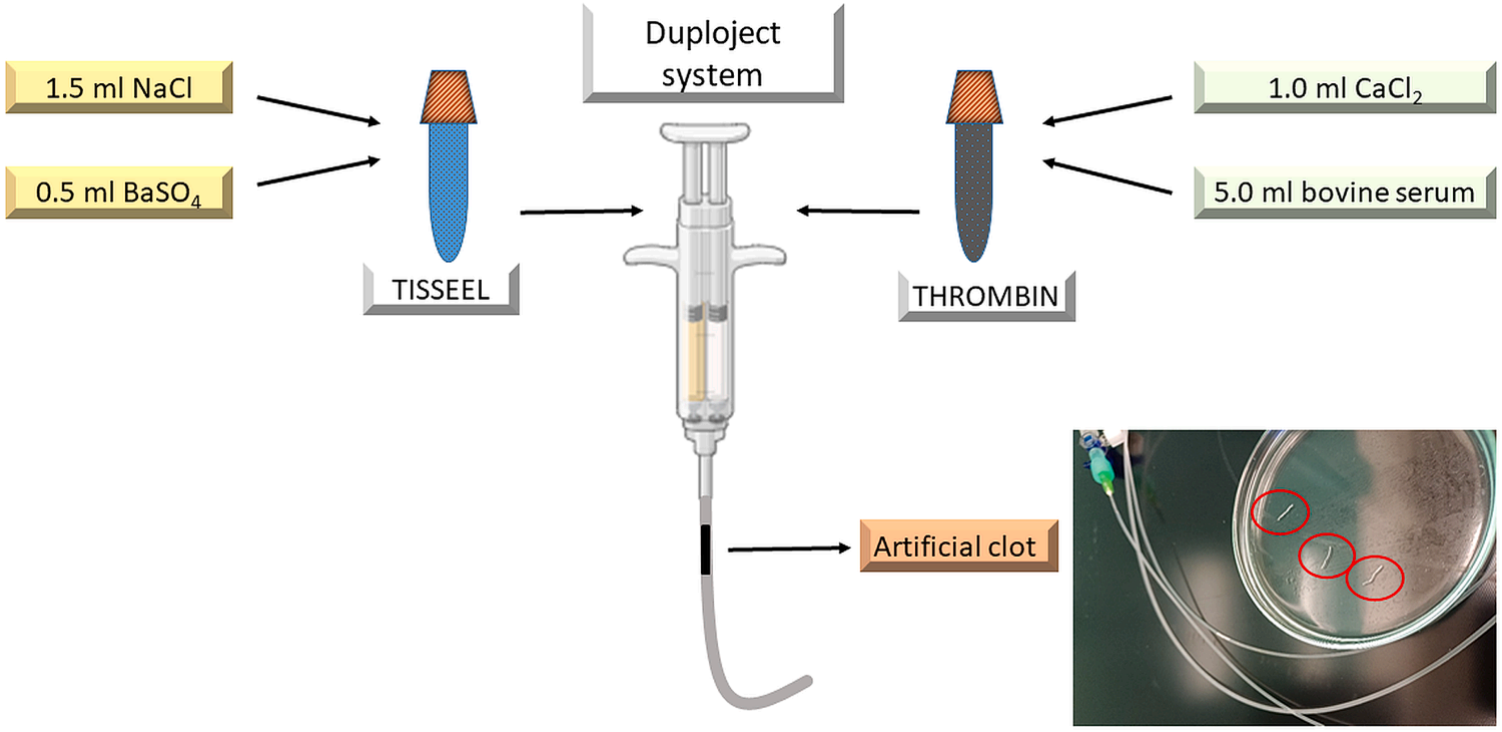
Scheme of artificial clot preparation using TISSEEL kit and Duploject System. Created using PowerPoint and icon of BioRender.com.

**Fig. 2:**
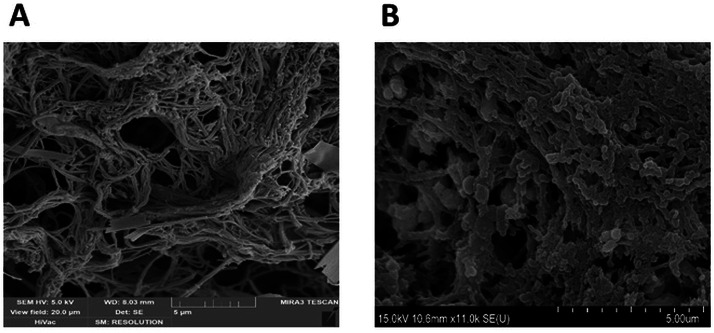
Microscopic clot structure. Representative images of the artificial clots used in systemic embolization model in rats under scanning electron microscopy. A - Fibrin network of artificial clot without barium sulfate B - Fibrin network of artificial clot labeled with barium sulfate.

Commercial human fibrin glue, the TISSEEL kit (Baxter, USA) was used as the blend basis for the artificial clot containing 60 % of fibrin [18]. Saline solution (NaCl, 1.5 ml) and barium sulphate (BaSO_4_, 0.5 ml) (Micropaque, Guerbet) we applied into the TISSEEL ampoule. Then 1 mL of calcium chloride (CaCl_2_) solution and 5 ml of bovine serum were applied into the “Thrombin 500 I.U.” ampoule included in the kit. Using the original Duploject system (Baxter, USA, Fig. 1), the solutions from Tisseel and Thrombin 500 IU ampoules were injected into PE60 tubes (Braintree Scientific, USA), at equal parts. The PE60 tubes were closed on both ends with silicone (prevents desiccation) and incubated at 37 °C for 120 min. Then, the coagulated artificial clots were stored 24 h at 4–8 °C. The next day, the clot was cut into pieces of 10 mm in length and displaced to Eppendorf tubes with saline solution (3 pieces per tube). All clots were stored at −20 °C for a maximum of 2 weeks. The microstructure of the artificial clot labeled with BaSO_4_ is shown in Figs. 2A and 2B . All artificial clots were prepared under identical conditions.

• *Anesthesia, catheter insertion, embolisation*

**Fig. 3:**
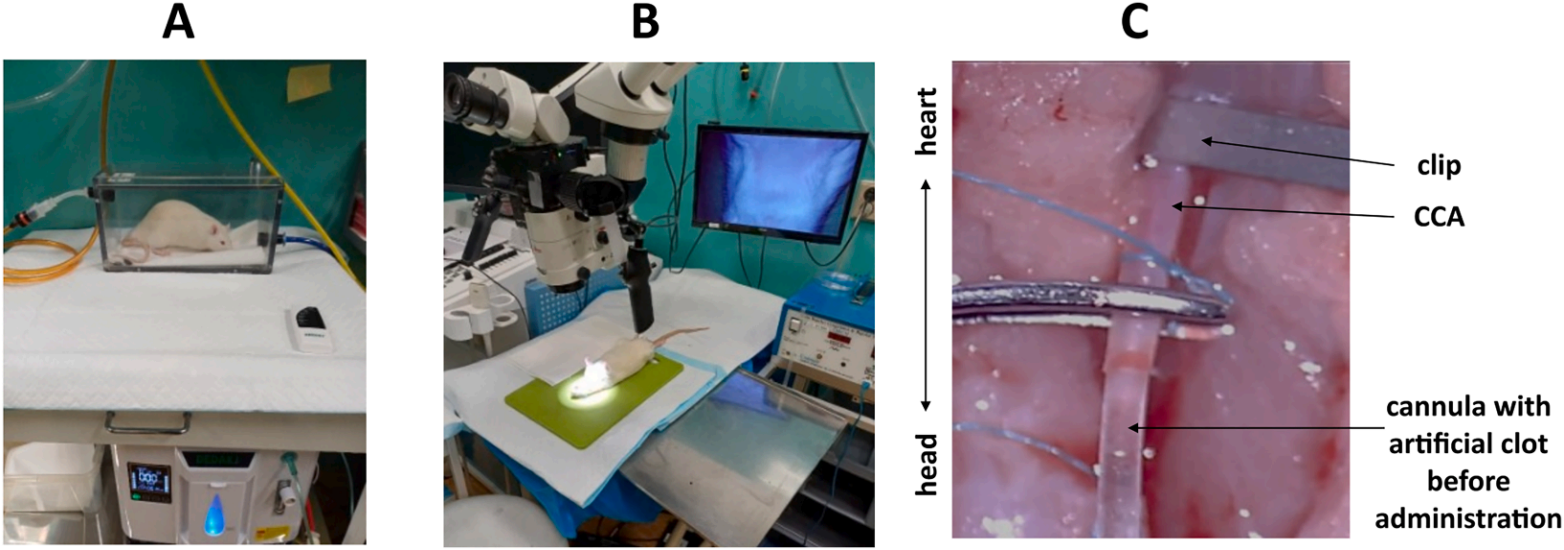
Anesthesia and preparation of surgery area for clot application. A - rat placed to plastic box for isoflurane inhalation anesthesia; B - surgery table and position of fully anesthetized animal before surgery procedure; C - visualization and ligation of common carotid artery (CCA) and external carotid artery (ECA) for clot application.

During all procedures, rats were under general anesthesia as described previously [19], induced by the initial inhalation of 2.5% isoflurane in the carrier gas (filtered room air, Fig. 3A). During the isoflurane induction, xylazine and ketamine were injected intramuscularly (5 mg/kg and 35 mg/kg of bodyweight, respectively), and a mixture of urethane (80 mg/ml) and alpha-chloralose (6 mg/ml) in a dose of 0.8 ml per 100 g of body weight was applied into the peritoneal space. Fully anesthetized animal was placed on the surgery table (Fig. 3B). Subsequently, the left common carotid artery (CCA) was isolated, visualized, and cannulated with a PE50 cannula (Braintree Scientific, USA) in retrograde towards the aortic arch with a distal ligation (at the head end) and temporarily clamped proximally end (at the heart end) using a micro-bulldog clamp (Fig. 3C).

**Fig. 4:**
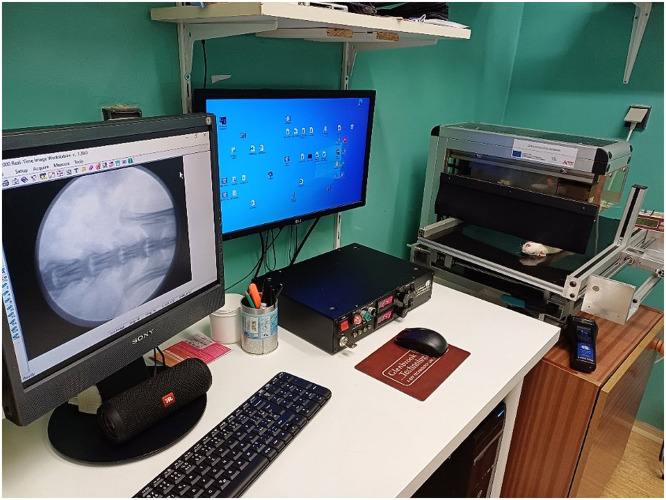
Animal positioned under the C-arm of micro-fluoroscopy system and visualization of the abdominal cavity.

Using the PE50 catheter, three artificial clots were injected into the aortic arch. After cannula removal, the left common carotid artery was permanently ligated, and the wound was closed in layers. The tail vein was cannulated (26 G Surflo-W, Terumo, Germany) and connected to a syringe pump (PPS9001S, Onyx, CZ) for rtPA or saline solution infusion. Using the C-arm micro-fluoroscopy system, the check of the position of the artificial clots in the branches of the abdominal aorta was performed (Fig. 4).

Then, the animals were positioned for better visibility of most of the clots, with the maximum area of their size, and ideally without shielding of bone structures.

The same procedure with animals (anesthesia, clot application, imaging) was performed using the micro-CT Skyscan 1276 and also with a preview scan (2D) for clots detection. Then the central camera was positioned, using an Aluminium/Copper filter, camera binning 1008 × 672 px, image pixel size 42.2 μm and rotation over 180 degrees, and scanning started. Each scan lasted 46 s and contained 325 projections.

For both imaging methods, the first image at time zero (before starting the infusion of rtPA/saline) was acquired. The time between the clot insertion and the infusion start was 10 min. Subsequently, an infusion of rtPA or saline solution from a syringe pump (A400, USA; flow rate of 1.3–-1.5 ml/hour ) started, and consecutive images (radiographs) were acquired every 10 min until the 60th minute of the experiment protocol. At the end of the imaging session, all animals were euthanized by decapitation under general anesthesia. Imaging data acquired by micro-fluoroscopy were blinded for evaluators.

• ***Clot lysis outcome***

**Fig. 5:**
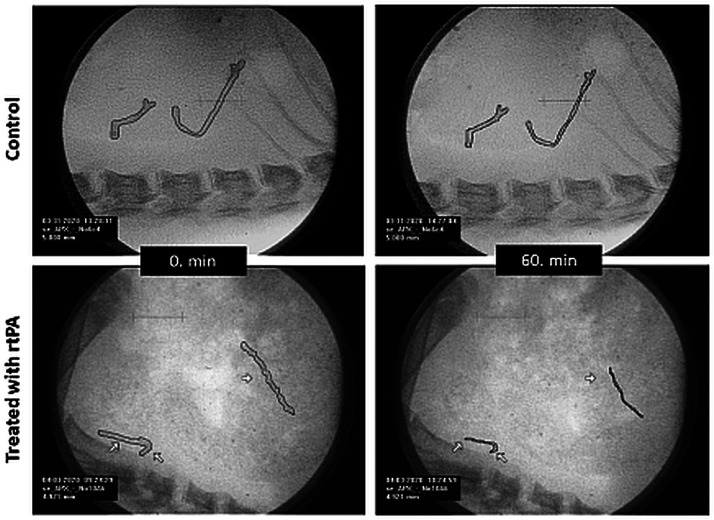
Radiographs with shadows of the artificial clots in control (upper images) and 0.9 rtPA treated group (lower images). An example of ImageJ shadow area measurement at the beginning (0 min) and the end of the experimental protocol (60 min) is shown per each group.

The outcome was measured as a relative change in the area of artificial clots, and the lysis rate was then calculated over time. The micro-fluoroscopy radiographs were analyzed in ImageJ 1.52a software (Wayne Rasband, National Institutes of Health). After the calibration setup, the contour of the area of the artificial clot was manually drawn, and the size in pixels was recorded (see Fig. 5).

**Fig. 6:**
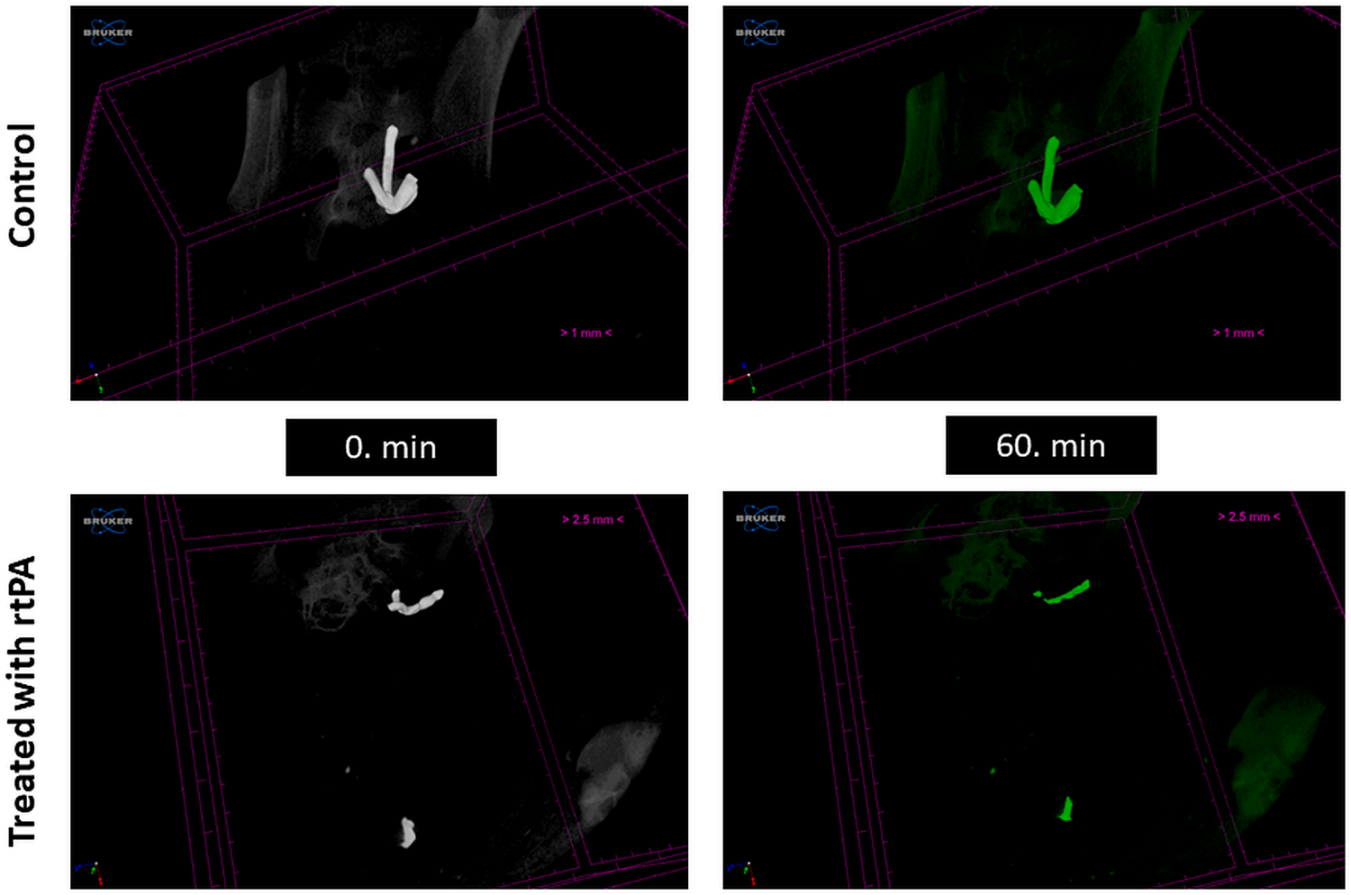
3D volume rendering visualization of the artificial clots in control (upper images) and 0.9 rtPA treated group (lower images). An example of clot size measurement at the beginning (0 min, white clots) and the end of the experimental protocol (60 min, green clots) is shown for both groups.

The projection dataset acquired from micro-CT was reconstructed using N-Recon software version 2.0 (Bruker micro-CT, Belgium) with a post-alignment correction, ring artifact correction (level 5), and no beam hardening reduction. The reconstructed dataset was processed using CTAn software (Bruker Micro-CT Software, Belgium). First, a region of interest containing the entire volume of clot was created manually for each scan. The rest of the 3D image analysis was performed exactly with the same settings for every single scan and animal. The threshold was set up to a value of 110 at the grayscale index and 3D image analysis was performed using a custom processing function of CTAn software. Using micro-CT, the total volume of the artificial clot was measured in cubic millimeters. Thereafter, the image dataset was saved as a bitmap, and 3D volume rendering visualization was performed in CTVox software (Bruker Micro-CT Software, Belgium) (Fig. 6). The same visualization was used for an artificial clot in the brain in the sensitivity analysis; see Supplementary Fig. S1.

The change in the clot area (%, change of clot volume in micro-CT imaging) was calculated versus the size of the clot at baseline before rtPA or saline solution administration (area of the clot at 0. minute – clot area at calculated minute) *-100 / area of the clot at 0. minute). The fibrinolysis rate (lysis reported in %/min) represents a relative change in the area (relative change in the clot volume in micro-CT imaging) of the artificial clot divided by the minute in which the area was measured (relative change of area of the artificial clot % / a minute in which the clot was measured).

The image analysis (for micro-fluoroscopy radiographs only) was performed by 2 persons blinded to each other’s readings and to animal group allocation. The protocol for image processing and clot measurement was as follows: 1) the images were enlarged by 200%, 2) the clots were numbered from left to right and top to bottom, and their area was measured in pixels, 3) if the area of two clots in different arteries overlapped, the size was measured as a single clot, 4) if the clot moved due to lyses off from view, only available data were used to the following analyses (calculation of change of clot area and lysis rate), 5) if the lysis of the clot caused moving of the clot but still visible on the image, the shadow area of the clot was included in the analyses, 6) all images of one animal were measured in one reading sequence at once, and 7) after all measurements, the anatomical location of the clot in the respective artery was determined, as well as the appropriate animal group.

• ***Statistics***

The parameters used for validation of thrombolytic efficacy of rtPA in the systemic embolization (SE) model included relative change in clot area (or clot volume for micro-CT imaging; %), lysis rate (%/min), and area under the thrombolytic curve (AUC; %*min) for both imaging modalities. These parameters were selected to capture complementary aspects of thrombolysis dynamics and to support construct validity through consistent measurement of the underlying biological process. For each group and time point, results were expressed as mean ± standard deviation (SD). Thrombolytic curves were generated from temporal changes in clot size, and AUC was calculated to provide an integrated measure of thrombus resolution over time.

To evaluate predictive validity and model sensitivity, differences between treatment groups (control, 0.9 mg/kg rtPA, and 5 mg/kg rtPA) were assessed using marginal models (among the clots from one individual animal) accounting for intra-subject clustering, as multiple clots were measured within individual animals. These models were used to estimate treatment effects on clot size reduction and lysis rate at predefined time points (10, 30, and 60 min), as well as across the full experimental time course.

Concurrent validity was assessed by comparing thrombolytic parameters (relative clot change, lysis rate, and AUC) obtained using micro-fluoroscopy and micro-CT imaging. Agreement between methods was evaluated by comparing mean differences and corresponding 95% confidence intervals across time points using the unpaired two sample t-test assuming normality of data and equal variances or the non‑parametric Mann–Whitney U test.

Reliability of image-based measurements was evaluated using the intra-class correlation coefficient (ICC), calculated with a two-way mixed-effects model for absolute agreement (k = 2 evaluators), with 95% confidence intervals.

To characterize variability and support reproducibility assessment, inter-individual and intra-group variability were quantified using the coefficient of variation (CV), based on clot size changes at 60 min.

Sample size was estimated based on expected effect size from prior thrombolysis studies [20,21], assuming a 63% reduction in clot size between rtPA-treated and control groups with a standard deviation of approximately 40%. At a significance level of α = 0.05 and power of 80%, a minimum of 6–8 animals per group was required. To account for intra-animal clustering and potential attrition, 9–10 animals per group were included. The observed treatment effects were consistent with the assumptions used for sample-size planning and provided sufficient precision to detect clinically relevant differences between groups.

To assess the robustness of the model across anatomical conditions, subgroup analyses were performed comparing clot lysis in terminal versus non-terminal arteries [22]. Differences were evaluated using the same marginal modeling framework.

All analyses were performed using GraphPad Prism 8.4.2 (GraphPad Software, USA) and SPSS version 23 (IBM Corp., USA). A two-sided p-value <0.05 was considered statistically significant.

During image analysis, transient changes in clot size due to physiological motion (e.g., respiration or intestinal movement) were identified. Measurements affected by complete loss of clot visibility were excluded based on predefined criteria, and analyses were repeated to confirm robustness of results (see Supplementary Fig. S3).

## Method validation

Of 51 animals, 49 were included in the final analyses. Two animals randomized to the 5 rtPA treated group were excluded from the analyses due to unsuccessful cannulation. Of the 84 and 63 clots applied and screened by micro-fluoroscopy and micro-CT, respectively, 55 and 36 were included in the final analyses.

### The effect of thrombolysis in treated groups versus control – micro-fluoroscopy

**Fig. 7:**
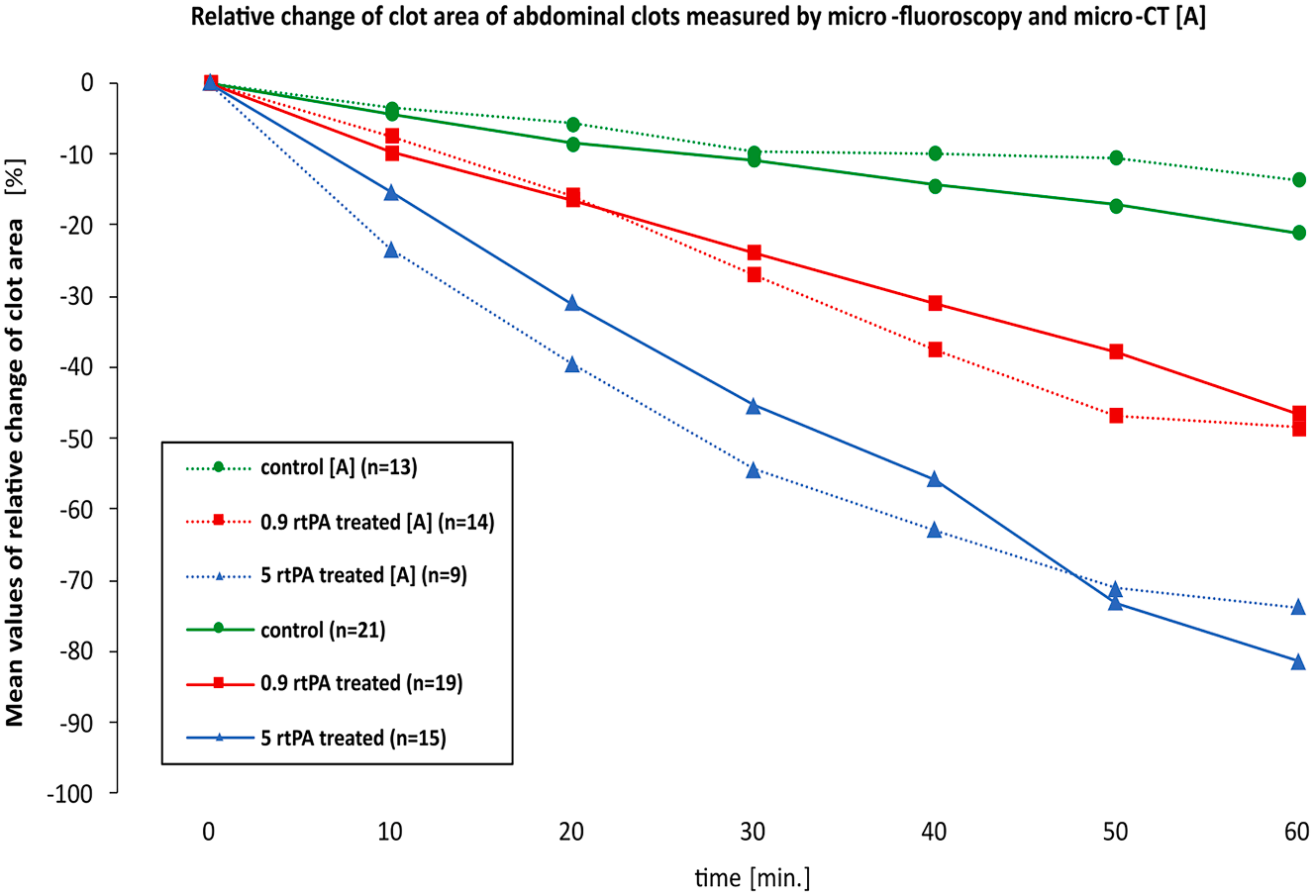
The average relative change in the area or clot volume (mean %) of artificial clots in control (green) and 0.9 rtPA (red) and 5 rtPA (blue) treated groups of animals within 60-minute protocol, measured by micro-fluoroscopy and micro-CT imaging. The graph shows the lytic effect of alteplase initiating at 10 min of intravenous thrombolysis. [A] = values acquired by micro-CT imaging; n = number of clots..

The thrombolytic effect was observed shortly after administration of rtPA. The difference in the average relative change in the area of artificial clots (%) through the whole experiment in the 0.9 and 5 rtPA treated and control groups is shown in Fig. 7. Starting at 10 min, the clot area in the both treated groups changed significantly compared to the control group and the difference in the clot size increased over time in both treated groups. The relative change in the area of the clots (%) between the 0.9 rtPA treated and control group revealed a detectable decrease in size by −9.7 ± 7.4 vs. −4.3 ± 7.0 at 10 min (adjusted absolute difference: 5.4; 95%CI [0.7 to 11.5]) and −46.4 ± 25.7 vs. −21.1 ± 14.3 at 60 min (adjusted absolute difference: 25.3; 95%CI [6.5 to 44.2]). Comparison of the relative change in the area of the clot between the 5 rtPA treated and control group revealed a detectable decrease in size by −15.3 ± 9.7 vs. −4.3 ± 7.0 at 10 min (adjusted absolute difference: 11.0; 95%CI [4.5 to 17.5]) and −81.3 ± 21.9 vs. −21.1 ± 14.3 at 60 min (adjusted absolute difference: 60.2; 95%CI [41.6 to 78.9]). Dose dependent thrombolytic efficacy of rtPA was also found in CoWBOi model in the sensitivity analysis, see Supplementary Fig. S2.

Treatment with both doses of rtPA caused a detectable difference between groups, the average lysis rate (%/min) in the 0.9 rtPA treated vs. control group was as follows: 1.0 ± 0.7 vs. 0.4 ± 0.7 within 10 min (adjusted absolute difference: 0.5; 95%CI [0.1 to 1.1]) from the start of the experiment, 0.9 ± 0.6 vs. 0.4 ± 0.4 within 30 min (adjusted absolute difference: 0.5; 95%CI [0.0 to 0.9]), and 0.8 ± 0.5 vs. 0.4 ± 0.3 within 60 min (adjusted absolute difference: 0.4; 95%CI [0.1 to 0.8]). The average lysis rate (%/min) in the 5 rtPA treated vs. control group was as follows: 1.5 ± 1.0 vs. 0.4 ± 0.7 within 10 min (adjusted absolute difference: 1.1; 95%CI [0.5 to 1.7]) from the start of the experiment, 1.3 ± 0.7 vs. 0.4 ± 0.4 within 30 min (adjusted absolute difference: 0.9; 95%CI [0.5 to 1.4]), and 1.4 ± 0.6 vs. 0.4 ± 0.3 within 60 min (adjusted absolute difference: 1.0; 95%CI [0.6 to 1.4]).

The obtained ICC value for relative change in the clot area was 0.89 (95% CI 0.67 to 0.95). The level of reliability between two evaluators is good to excellent [23].

The area under the thrombolytic curve (AUC; %*min) was calculated as the second parameter describing the change in thrombus size over time. AUC in the time 0 to 60 min was -657 ± 451 in the control group vs. -1418 ± 824 in the 0.9 rtPA treated group, adjusted absolute difference: 761; 95%CI [157 to 1366]. AUC in the time 0 to 60 min was -657 ± 451 in the control group vs. -2613 ± 1079 in the 5 rtPA treated group, adjusted absolute difference: 1956; 95%CI [1310 to 2602].

### Comparison of the effect of thrombolysis in treated and control groups using micro-fluoroscopy versus micro-CT

**Fig. 8:**
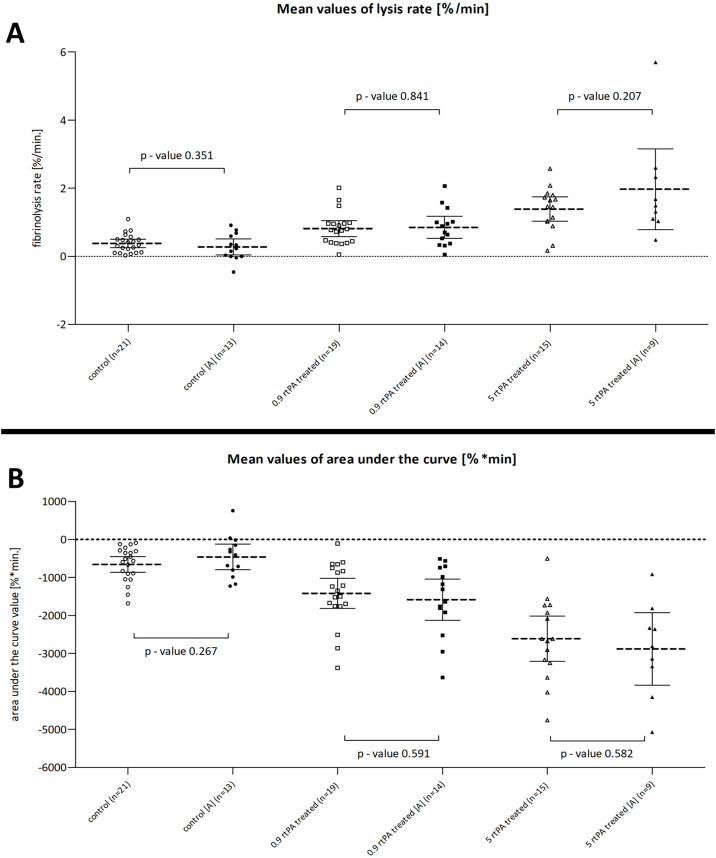
Comparison of lysis rate (mean %/min; A) and area under the curve (mean%*min; B) within 60 min of experiment detected, using micro-fluoroscopy and micro-CT. [A] = values acquired by micro-CT imaging; rtPA = alteplase; n = number of clots. The error bars represent 95% CI of the mean..

The detection of thrombolytic effect using the microCT was comparable to fluoroscopy imaging (Fig. 7). No differences in lysis rate (Fig. 8A) or AUC (Fig. 8B)were observed between micro-fluoroscopy and micro-CT within the corresponding treatment and control groups.

No significant difference was found in the relative change in clot area or volume at any time point when comparing micro-fluoroscopy with micro-CT measurements, see Table 1.

**Table 1:** Comparison of thrombolytic efficacy of alteplase (rtPA) in the systemic embolization model presented as adjusted absolute difference in average change in clot area or volume between 0.9 rtPA (a), 5 rtPA (b) treated and control (c; saline solution) groups detected by micro-fluoroscopy versus micro-CT imaging. No difference was found when thrombolytic effect was scanned by micro-fluoroscopy or micro-CT. [A] = values acquired by micro-CT imaging; n = number of clots.

	a) Efficacy of thrombolysis by 0.9 mg/kg rtPA using micro-fluoroscopy vs. micro-CT [A]			b) Efficacy of thrombolysis by 5 mg/kg rtPA using micro-fluoroscopy vs. micro-CT [A]			c) Efficacy of thrombolysis by saline solution using micro-fluoroscopy vs. micro-CT [A]		
	Average relative change in the clot area (%; mean ±SD)			Average relative change in the clot area (%; mean ±SD)			Average relative change in the clot area (%; mean ±SD)		
Group/time (min.)	0.9 rtPA treated (n=19)	0.9 rtPA treated [A] (n=14)	Adjusted absolute difference and 95%CI	5 rtPA treated (n=15)	5 rtPA treated [A] (n=9)	Adjusted absolute difference and 95%CI	control (n=21)	control [A] (n=13)	Adjusted absolute difference and 95%CI
10.minutes	−9.7 ± 7.4	−7.3 ± 8.9	−2.3 (−8.1 to 3.5)	−15.3 ± 9.7	−23.3 ± 24.6	8.1 (−6.6 to 22.7)	−4.3 ± 7.0	−3.5 ± 9.6	−0.8 (−6.7 to 5.1)
20.minutes	−16.4 ± 11.3	−15.8 ± 14.4	−0.6 (−9.7 to 8.6)	−31.1 ± 22.8	−39.4 ± 24.3	8.4 (−12.1 to 28.8)	−8.5 ± 8.7	−5.6 ± 14.5	−2.9 (−10.9 to 5.2)
30.minutes	−23.9 ± 15.5	−26.9 ± 15.6	3.1 (−8.1 to 14.2)	−45.4 ± 26.5	−54.3 ± 25.8	8.9 (−14.0 to 31.8)	−10.8 ± 8.2	−9.8 ± 9.8	−1.1 (−7.4 to 5.3)
40.minutes	−30.9 ± 18.5	−37.4 ± 22.2	6.5 (−8.0 to 20.9)	−55.8 ± 28.6	−63.0 ± 24.9	7.2 (−16.6 to 31.1)	−14.4 ± 10.2	−9.8 ± 10.3	−4.6 (−12.0 to 2.8)
50.minutes	−37.8 ± 21.1	−46.8 ± 26.0	9.0 (−7.7 to 25.8)	−73.1 ± 25.4	−71.2 ± 23.8	−1.9 (−23.7 to 19.8)	−17.2 ± 14.0	−10.6 ± 13.1	−6.6 (−16.4 to 3.2)
60.minutes	−46.4 ± 25.7	−48.5 ± 25.9	2.1 (−16.5 to 20.6)	−81.3 ± 21.9	−73.7 ± 25.6	−7.6 (−27.9 to 12.8)	−21.1 ± 14.3	−13.5 ± 9.6	−7.5 (−16.7 to 1.6)

### Analysis of clot location in rtPA treated and control groups – micro-fluoroscopy

**Fig. 9:**
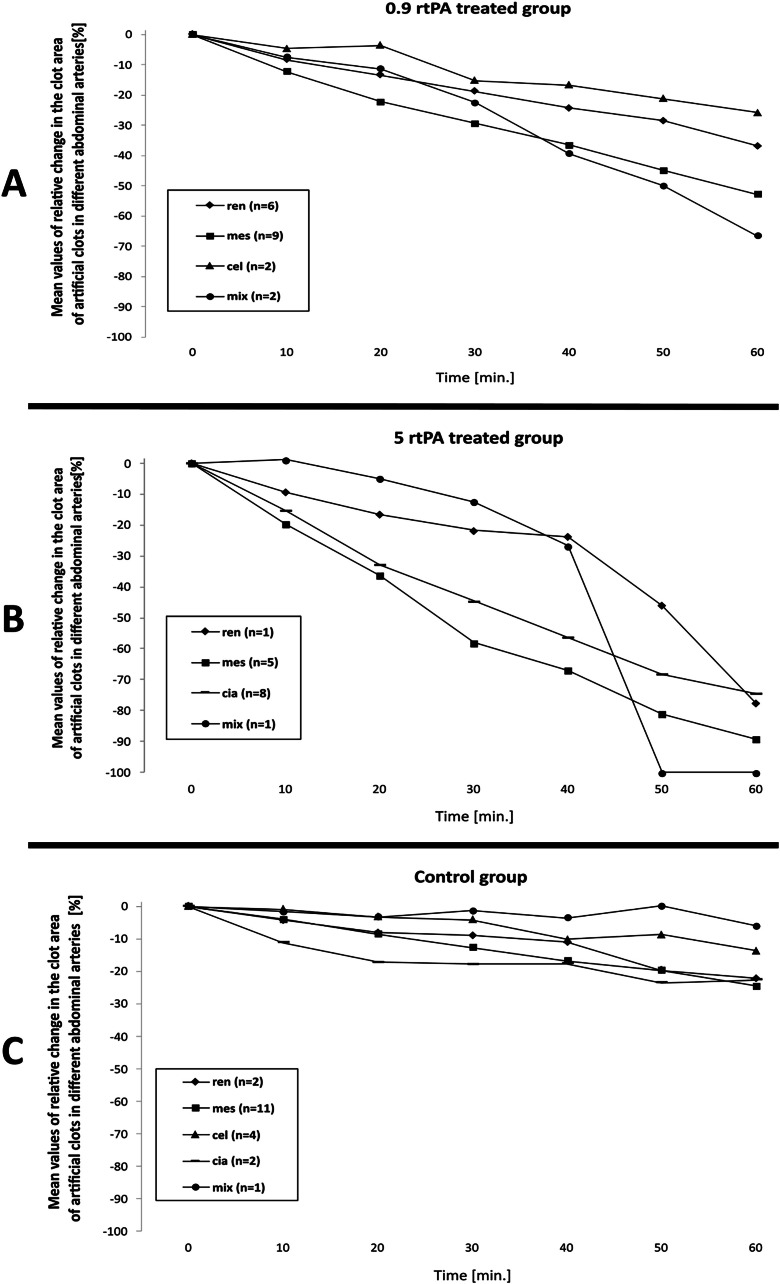
The comparison of lysis curves in different arteries in all groups of rats using micro-fluoroscopy. The average (mean) relative change in the area (%) of artificial clots in different arteries in 0.9 mg/kg rtPA treated (A), 5 mg/kg rtPA treated (B), and control (A; with spontaneous thrombolysis) groups within the 60-minute protocol. REN= a. renalis; MES = a. mesenterica; CEL = a. celiaca; CIA = a. iliaca communis; MIX = mix of arteries (two or three clots overlapped, analysed as one clot without artery type determination); n = number of clots.

The average relative change in the area of artificial clots (%) for each artery in 0.9 rtPA (19 clots in 8 terminal arteries, 9 non-terminal arteries, and 2 without artery type determination, Fig. 9A), 5 rtPA (15 clots in 1 terminal artery, 13 non-terminal arteries, and 1 without artery type determination, Fig. 9B) and the control group (21 clots in 6 terminal arteries, 14 non-terminal arteries, and 1 without artery type determination, Fig. 9C) is shown in Fig. 9, demonstrating that the thrombolytic effect was similar regardless of its location in the artery. The analysis of the average lysis rate aimed on artery type showed that the lytic effect on the clot was not influenced by the localization (control group p = 0.27; treated group p = 0.14), therefore the model was not adjusted for artery location.

There was no effect of animal or group, the inter-individual and intra-group variability was 47% (95%CI 39 to 54) and 46% (95%CI 40 to 51) in the control group, respectively; and 62% (95%CI 47 to 76) and 46% (95%CI 32 to 60) in the treated group, respectively.

A power analysis of the relative change in the clot area after the study showed that the sample size of 7 clot pairs has 80% power to detect an effect size of 25.3% assuming a 5% significance level. The sample size analysis for lysis rate revealed minimally 6, and for AUC, minimally 7 clot pairs. Thus, the number of animals and applied clots in each group, and for each parameter tested, was sufficient.

### Imaging usability and model safety

Using the micro-fluoroscopy imaging, of 84 artificial clots applied, 7 rats presented 3 clots, 13 rats presented 2 clots, and 8 rats presented 1 artificial clot in abdominal aorta branches. Micro-fluoroscopy imaging revealed a total of 21 clots in 10 control animals and 34 clots in 18 animals of both treated groups. Of all animals, the capturing one, two, and three measurable clots per animal was 100%, 71%, and 25%, respectively. All animals presented at least one clot, with a mean number of 2 clots per animal. During the monitoring period, no complications or mortality were observed. The distribution of artificial clots in the branches of the abdominal aorta was as follows: renal artery 9 (16%), celiac artery branches 6 (11%), iliac artery branches 11 (20%), cranial mesenteric artery branches 25 (45%), and mix of arteries 4 (7%). The shadows of two artificial clots overlapped, so it was analysed as one clot without artery type determination (referred to as “mix”). In five animals from the 0.9 rtPA treated group, part of the clot moved transversally, so only the part visible in all images was included in the analyses. Due to the distal embolization of three clots in the treated group at 40 and 55 min outside the field-of-view, the images were excluded. For the results of analysis with uncovered and excluded clots, see Supplemental Material.

In the SE model with micro-CT imaging, of 63 artificial clots applied to 21 rats (3 per animal), 3 rats presented 3 clots, 9 rats presented 2 clots, and 9 rats presented 1 artificial clot in abdominal aorta branches. By micro-CT imaging, a total of 13 clots in 7 control animals, 14 clots in 7 animals of 0.9 rtPA treated and 9 clots in 7 animals of 5 rtPA treated groups were detected. Of all animals, the capturing one, two, and three measurable clots per animal was 100%, 57%, and 14%, respectively. All animals presented at least one clot, with a mean number of 2 clots per animal. During the experimental period, no complications or mortality were observed.

## Limitations

Despite its advantages, the presented model has several limitations. First, C-arm micro-fluoroscopy provides two-dimensional imaging, whereas micro-CT enables three-dimensional visualization and direct clot volume measurement. Although micro-CT was used as a reference method and showed comparable thrombolytic metrics, micro-fluoroscopy relies on projected clot area as a surrogate measure and does not provide volumetric data.

Second, micro-fluoroscopy and micro-CT imaging resulted in approximately 30% and 40% data loss, respectively, due to the restricted field of view, clot displacement outside the scan area, or by analysis of two indistinguishable clots as one when a single vessel is embolized. This limitation reduces detection efficiency compared with micro-fluoroscopy and highlights that, while quantitatively comparable, the two imaging modalities differ in practical usability and coverage. Micro-fluoroscopy is therefore more suitable for dynamic, whole-abdomen monitoring, whereas micro-CT is better suited for targeted validation.

Third, some clots changed position or moved partially out of the imaging field during thrombolysis, requiring predefined exclusion criteria. Although sensitivity analyses confirmed that these exclusions did not materially affect the results, they underscore the importance of standardized imaging protocols and careful interpretation of dynamic clot behaviour.

Fourth, while the use of multiple clots per animal improves experimental efficiency, clots within a single subject are not fully independent. Although statistical models accounting for intra-animal clustering were applied, this dependency should be considered when interpreting results. Fifth, in the present experiment, we did not focus on the blood flow restoration during the thrombolysis. However, micro-fluoroscopy enables this process with compatible contrast media (e.g., iodine-based).

Finally, the SE model is not designed to assess thrombolysis-associated hemorrhagic complications or tissue-level ischemic injury. For studies focused on safety outcomes, hemorrhagic transformation, or neurological function, cerebral ischemia models such as middle cerebral artery occlusion remain more appropriate.

Despite these limitations, the SE model fulfills its intended role as a pharmacodynamic screening platform and provides a robust, practical, and quantitatively validated method for studying thrombolysis.

## Ethics statements

All procedures and animal experiments were performed in full compliance with the ARRIVE and the European Community Council Directive (2010/63/EU) for Protection of Vertebrate Animals Used for Experimental and other Scientific Purposes guidelines. The project was approved by the Institutional Committee for the Protection of Experimental Animals of Masaryk University Brno and by the Committee for the Protection of Experimental Animals of the Ministry of Education Youth and Sports in the Czech Republic (MEYS CZ) with protocol numbers MSMT-35804/2019–2 and by The Ministry of Agriculture of the Czech Republic with the protocol number MZe 2162.

## Related research article

None.

## CRediT authorship contribution statement

**Peter Scheer:** Conceptualization, Funding acquisition, Investigation, Methodology, Resources, Validation, Writing – original draft. **Jana Hložková:** Investigation, Methodology, Resources, Writing – original draft. **Adam Novobilský:** Data curation, Investigation, Methodology, Visualization, Writing – original draft, Writing – review & editing. **Jaroslav Ondruš:** Writing – original draft, Writing – review & editing. **Eliška Brhelová:** Data curation, Formal analysis, Resources, Visualization. **Jana Doležalová:** Formal analysis, Visualization, Writing – original draft, Writing – review & editing. **Ahmet Davut Aksu:** Investigation, Resources. **Radka Opatřilová:** Writing – original draft, Writing – review & editing. **Alba Grayston:** Writing – original draft, Writing – review & editing. **Anna Rosell:** Writing – original draft, Writing – review & editing. **Josef Mašek:** Funding acquisition, Writing – review & editing. **Robert Mikulík:** Funding acquisition, Project administration, Supervision, Writing – original draft, Writing – review & editing.

## Declaration of competing interest

The authors declare that they have no known competing financial interests or personal relationships that could have appeared to influence the work reported in this paper.

## Data Availability

Data will be made available on request.

## Appendix Supplementary materials

Image, application 1
