# Supplementary material for: A Systemic embolization model in a rat with real-time micro-fluoroscopy for direct quantification of thrombolysis
Source: MethodsX. 2026 Jul 7;17:104036. doi: 10.1016/j.mex.2026.104036 (PMC13453478; doi:10.1016/j.mex.2026.104036)
Supplement: Supplementary file 1 [file mmc1.docx]

**Supplementary material *and/or* additional information [OPTIONAL]**

**1. Circle of Willis embolization model with micro-CT imaging**

*Experiment design*

To evaluate the thrombolytic efficacy of alteplase (rtPA) in dose of 0.9 mg/kg/h and 5 mg/kg/h as used in systemic embolization model (SE model), the COWBOi (**C**ircle **O**f **W**illis **B**ranches **O**cclus**i**on) model with human-fibrin based clots was chosen. The thrombolysis was detected by the micro-CT Skyscan 1276 with the same calibration and values as in SE model (see Main text). Male outbreed Wistar rats (n = 24; bodyweight 330-350 g) were randomly divided into three groups as follows: 1/ 0.9 mg/kg rtPA (n = 8); 2/ 5 mg/kg rtPA (n = 8) with an initial bolus of 10% volume and 90% infusion for 60 min. (a flow rate 1.3 - 1.5 ml/hour); and 3/control group (n = 8) with the saline solution in equivalent volume and time.

The including criteria were the presence of clots in the preview scan localized in branches of the Circle of Willis and a functional intravenous cannula.

Excluding criteria were death before the clot application or death before the 60th minute of rtPA/saline infusion. All animal experiments were performed under the Czech (law Nr. 246/1992) and EU legislation (EU Directive 2010/63/EU) and were approved by the government authority (ethical permit ID: MZe 2162). All procedures, including animal design, were in agreement with ARRIVE guidelines.

*Clot preparation, anesthesia, rtPA treatment and micro-CT scanning*

Barium sulphate-labeled human-fibrin based clots were prepared as for the SE model. Before the application to the carotid artery of rats, the clots were warmed up to laboratory temperature and cut to acquire a 3 mm length.

During the COWBOi model experiment, the animals were under general anesthesia, initiated with 2.5% isoflurane, followed by administration of xylazine together with ketamine (i.m., 5 mg/kg and 35 mg/kg BW, respectively), a mixture of urethane (80 mg/ml) and alpha-chloralose (6 mg/ml) in a dose of 0.8 ml per 100 g of body weight was administered intraperitoneally, as described for SE model.

The animal was placed on a heating plate (40°C) in its back position. After shaving and disinfecting of ventral side of the neck, the skin incision was performed in the medial line in a supine body position. Left common carotid artery (CCA) with its branches, external carotid artery (ECA), and internal carotid artery (ICA), were separated from nerves and connective tissue, and arterial branches of *ramus thyroideus* and *a. occipitalis* were cauterized with the bipolar electrosurgical device (Malis Bipolar Cutter, Codman and Shurtleff Inc.). The pterygopalatine artery was ligated (7-0 USP monofilament silk). Then ECA was ligated distally (=rostral) by USP 6-0 monofilament suture and beneath this ligature (=proximally) a second ligation was performed using USP 7-0 monofilament suture. The ICA and CCA were also occluded temporarily. The ECA was cut between 2 ligatures and catheterized by PE10 cannula (Braintree Scientific) retrogradely. The cannula was moved through ECA also to ICA rostrally. The catheter was fixed with a double suture using USP 7-0 monofilament suture. Via PE 10 catheter (Braintree Scientific), two artificial clots 3 mm long prepared in PE10 cannula were applied into the ICA. The surgical wound was closed by suture. To avoid drying, the wound was washed with tempered (body temperature) saline solution continuously. Subsequently, the tail vein was cannulated (26 G Surflo-W, Terumo) and connected to a syringe pump for later rtPA or saline solution infusion.

Following clot arterial occlusion, rats were kept under anesthesia cannulated and transferred into scanning bed. Two hours after Circle of Willis embolization, animal brains were first scanned at micro-CT Skyscan 1276 to visualize the presence of clot (time 0). The initial bolus of rtPA/saline was applied via an intravenous cannula and the remaining rtPA/saline was administered as described above. Rat brains were scanned every 10 min. for an hour as described for the SE model and then the animals were euthanized.

*Data processing and analysis of micro-CT image datasets*

The projection images were acquired and analyzed as described for SE model using N-Recon software version 2.0 (Bruker micro-CT, Belgium) and the reconstructed dataset was processed using CTAn software (Bruker micro-CT, Belgium; Supplementary Fig. S1). The total volume of artificial clot was obtained in cubic millimeters. Thereafter, the image dataset was saved as a bitmap, and 3D volume rendering visualization was performed in CTVox software (Bruker micro-CT, Belgium).

**Supplementary Fig. S1**. Maximum intensity projections of micro-CT scanned rat heads containing visible barium sulphate labeled fibrin clot. Projections were composed of individual rat head originating from the control group (A), group treated with 0.9 mg/kg rtPA (B), and the group treated with 5 mg/kg rtPA (C). Scans were obtained at 0, 10, 20, 30 and 60 min. post treatment initiation; heads are visualized from ventral side. rtPA=alteplase.

*Statistical analyses*

Artificial clot volumes in time were expressed as real volume data and as volume reduction (Percentage of volume reduction = [clot volume at time 0 - clot volume at time X] × 100/clot volume at time 0). The clot volume reduction was analyzed using Graph-Pad Prism version 8.4.2. (Graph-Pad Software, USA). Prior to statistical analysis, data normality was assessed using the Shapiro-Wilk test, and homogeneity of variances was evaluated using the Brown-Forsythe test. One-way analysis of variance (ANOVA) followed by Dunnett’s and Tukey's multiple comparisons test was applied to assess differences in mean clot volume reduction between the three groups.

*Results*

Of 24 animals, 3 (12 %) were excluded. Two rats died (8 %) during the experiment before the start rtPA therapy, and in one rat (4 %) no clots were observed in Circle of Willis. Thus, clot was successfully administered in seven animals in each group and they were included in the study. There was no significant difference in initial clot volume between groups (Supplementary Fig. S2). Thrombolytic efficacy of alteplase was observed in two treated groups in the 10 min. of experimental protocol. In the 0.9 mg/kg rtPA and 5 mg/kg rtPA groups, complete lysis was detected for only one clot in 60 and 30 min., respectively, after the start of infusion therapy. There was no statistical difference between artificial clot mean volumes between the 0.9 mg/kg rtPA and 5 mg/kg rtPA groups at any time point. Real mean clot volumes differed significantly from the control group since 30 and 40 min. of experimental protocol in the 5 mg/kg rtPA and 0.9 mg/kg rtPA treated groups, respectively (Supplementary Fig. S2).

**Supplementary Fig. S2**. The clot lysis curve in 60 min. interval of recombinant tissue plasminogen activator therapy in 21 rats with Circle of Willis embolism using micro-CT imaging. The fibrinolysis rate is expressed as means ± 95% CI of real clot volumes (left plot) at 0, 10, 20, 30, 40, 50, 60 min. after the start of rtPA or saline treatment and as clot volume reduction in percentage (right plot). In each group, 7 clots were analyzed. Asterisk indicates significant difference (p<0.05) between treated and control group. rtPA=alteplase

**2. The effect of thrombolysis in control and treated groups in SE model after clot exclusions – micro-fluoroscopy**

After the clots exclusion, the relative change in the area of the clot (%) in the treated vs. control group decreased by -9.7 ± 7.4 vs. -4.3 ± 7.0 at 10 min. (adjusted absolute difference: 5.4; 95%CI [0.7 to 11.5]) and -46.4 ± 25.7 vs. -14.8 ± 8.4 at 60 min. (adjusted absolute difference: 31.6; 95%CI [13.6 to 49.7]). Comparison of the relative change in the area of the clot between the 5 rtPA treated and control group revealed a detectable decrease in size by -15.3 ± 9.7 vs. -4.3 ± 7.0 at 10 min., adjusted absolute difference: 11.0; 95%CI [4.5 to 17.5] and -74.5 ± 21.9 vs. -14.8 ± 8.4 at 60 min. (adjusted absolute difference: 59.7; 95%CI [38.1 to 81.4]) (Supplementary Fig. S3).

The average lysis rate (%/min) after clot exclusion was in the treated vs. control group 1.0 ± 0.7 vs. 0.4 ± 0.7 within 10 min. (adjusted absolute difference: 0.5 95%CI [0.1 to 1.1]) from the start of the experiment, 0.9 ± 0.6 vs. 0.4 ± 0.4 within 30 min. (adjusted absolute difference: 0.4; 95%CI [0.0 to 0.9]), and 0.8 ± 0.5 vs. 0.4 ± 0.4 within 60 min. (adjusted absolute difference: 0.4; 95%CI [0.0 to 0.8]). The average lysis rate after clot exclusion was in the 5 rtPA treated vs. control group 1.5 ± 1.0 vs. 0.4 ± 0.7 within 10 min. (adjusted absolute difference: 1.1 95%CI [0.5 to 1.7]) from the start of the experiment, 1.3 ± 0.7 vs. 0.4 ± 0.4 within 30 min. (adjusted absolute difference: 0.9; 95%CI [0.4 to 1.4]), and 1.4 ± 0.6 vs. 0.4 ± 0.4 within 60 min. (adjusted absolute difference:1.0; 95%CI [0.6 to 1.4]).

The area under the thrombolytic curve (AUC; %*min) slightly changed after the clot exclusion and in the time 0 to 60 min. it was -465 ± 286 in the control group vs. -1418 ± 824 in the treated group, adjusted absolute difference: 953; 95%CI [369 to 1537]. AUC in 5 rtPA treated group was
-1788 ± 1088 in the treated group, adjusted absolute difference: 1323; 95%CI [699 to 1945]. The obtained ICC value for lysis rate was after the clot exclusion 0.89 (95% CI 0.68 to 0.95).

**Supplementary Fig. S3.** Clot area changes over time after the clot exclusion. The average relative change in the area (%) of artificial clots in control (green), 0.9 mg/kg rtPA (red) and 5 mg/kg rtPA treated (blue) groups of animals within 60-minute protocol (with 95%CI for the mean). The graph shows the lytic effect of alteplase initiating at 10 min. of intravenous thrombolysis. rtPA=alteplase
